# Therapeutic cyproheptadine regimen in serotonin syndrome: Complications after cardiovascular surgery

**DOI:** 10.1002/ccr3.7720

**Published:** 2023-07-18

**Authors:** Ahmed Nagy, Aishah Nasir, Mahfujul Haque, Ramzan Judge, Joseph Lee

**Affiliations:** ^1^ Deborah Heart and Lung Center Browns Mills New Jersey USA; ^2^ Temple University Philadelphia Pennsylvania USA; ^3^ Michigan State University College of Human Medicine Grand Rapids Michigan USA

**Keywords:** cardiovascular surgery, drug interaction, methylene blue, postsurgery, serotonin syndrome

## Abstract

Serotonin syndrome can be a life‐threatening condition that occurs from the overactivity of serotonin in the central nervous system. This report describes the use of cyproheptadine for the management of serotonin syndrome in a patient taking fluoxetine and bupropion, who received methylene blue for vasoplegia syndrome. A 61‐year‐old female taking fluoxetine and bupropion preoperatively was given a total of three doses of methylene blue 100 mg IV within a brief time frame during and after a planned coronary artery bypass graft surgery. Postoperatively, the patient was not following commands, was agitated and confused, febrile with diaphoresis, tachycardic, had muscle rigidity, and horizontal ocular clonus. The patient's presentation was most consistent with serotonin syndrome due to a drug–drug interaction. Cyproheptadine and supportive care were used successfully to treat serotonin syndrome, and the patient was discharged home 14 days postoperatively. Based on the literature, there is no standardized method of weaning cyproheptadine when used for serotonin syndrome. The patient in our case received a total of 188 mg of cyproheptadine over the course of 10 days and did not experience any side effects. This case highlights a potential dosing regimen that can be used for other patients.

## 1 INTRODUCTION

Serotonin syndrome (SS) can be a life‐threatening condition that occurs from the overactivity of serotonin in the central nervous system. This syndrome has been seen with medication use (at therapeutic doses), accidental drug–drug interactions, and intentional medication overdose. Mechanisms by which agents can precipitate SS include increased serotonin production or release, impaired reuptake or metabolism, and direct agonism or increased sensitivity of the serotonin receptors (mainly 5‐HT1A and 5‐HT2A). The two most common drug classes implicated in SS are the selective serotonin reuptake inhibitors (SSRIs) and monoamine oxidase inhibitors (MAOIs).^1^

Clinical features of SS mainly include mental status changes, autonomic instability, and neuromuscular hyperactivity. Some pertinent physical findings will include hyperthermia, agitation, ocular/muscular clonus, mydriasis, tremor, hyperreflexia, muscle rigidity, diaphoresis, and increased bowel sounds.^1^ However, SS may vary in the presenting symptoms, making it a difficult syndrome to diagnose, which often means mild episodes are often missed, but severe episodes are often identified. SS is usually diagnosed using the Hunter Serotonin Toxicity Criteria.^1^, ^2^

When comparing SS to other similar differential diagnoses, SS tends to occur acutely after the insult happens and is usually short in its course, whether it is self‐limiting or requires treatment.^3^ The main aspects of care for SS include the discontinuation of insulting agent(s), supportive care which often includes sedation with benzodiazepines, administration of serotonin antagonists (e.g., cyproheptadine), and creating a plan to restart causative agent(s) after SS resolves (if still indicated).^1^

Vasoplegic syndrome is a condition with multifactorial pathophysiology which includes the activation of intrinsic vasodilatory pathways.^4^ Patients experience uncontrolled vasodilation and have limited response to vasopressors.^4^ Established treatment for vasoplegia following cardiac surgery include norepinephrine and vasopressin.^4^

Although cyproheptadine is often used as treatment in severe cases of SS, there is no standardized method of weaning doses once the medication is initiated. We report a case in which a cyproheptadine dosing regimen was used successfully, and without side effects, to treat SS caused by a drug–drug interaction with methylene blue in a female who underwent a planned coronary artery bypass graft surgery.

## 2 CASE PRESENTATION

A 61‐year‐old female with a history of premenstrual syndrome was taking fluoxetine and bupropion extended release for approximately 5 years. Other documented past medical history included Hodgkin's lymphoma status post doxorubicin treatment, hyperlipidemia, immune thrombocytopenia, tobacco dependence, asthma, and hemolytic anemia. The patient was admitted for worsening dyspnea and chest tightness. Cardiac catheterization demonstrated severe multivessel coronary artery disease. Further inpatient evaluation via transthoracic echocardiography found a depressed left ventricular ejection fraction (LVEF) of 30%–35% with severe inferior and inferolateral hypokinesis and moderate hypokinesis of the remaining walls, severely dilated left atrium, and moderate‐to‐severe mitral regurgitation with no mitral stenosis. Cardiothoracic surgery consultation recommended both coronary artery bypass graft (CABG) and mitral valve replacement. Home medications were stopped 2 days prior to surgery. During surgery, the patient was exposed to 121 min of aortic cross‐clamp time and 153 min of cardiopulmonary bypass time. They were volume expanded with 1.8 L of crystalloid fluid, 50 mL of albumin 25%, 1 unit of packed red blood cells, 2 units of fresh frozen plasma, 2 units of platelets, 20 units of cryoprecipitate, and 675 mL of cell saver. Coming off the pump, the patient was defibrillated twice, and an intra‐aortic balloon pump was inserted. After surgery, the patient experienced persistent and refractory vasoplegic syndrome and received a total of three doses of methylene blue 100 mg IV; two doses were given on postoperative day zero (POD#0; defined as day of surgery) and one dose was given on POD#1. Upon a medication reconciliation, it was also noted that one dose of meperidine 25 mg IV was administered to the patient on POD#0. On POD#1, the patient was extubated, but exhibited confusion and was not appropriately following commands. The patient was also agitated, hyperthermic (up to 39.9°C) with diaphoresis, tachycardic, had muscle rigidity, and horizontal clonus. The patient met the definition for SS based on the Hunter Serotonin Toxicity Criteria, including both the third and fourth scenarios listed in Table 1.^1^, ^2^ The presence of these symptoms was supportive of SS caused by a potential drug interaction between fluoxetine, bupropion, methylene blue, and meperidine.

**TABLE 1:** Hunter Serotonin Toxicity Criteria.^a^

Use of a serotonergic agent being administered in the past 5 weeks and one of the following scenarios: Tremor PLUS hyperreflexia Spontaneous clonus Muscle rigidity PLUS temperature > 38°C PLUS either ocular or inducible clonus Ocular clonus PLUS either agitation or diaphoresis Inducible clonus PLUS either agitation or diaphoresis

^a^ The Hunter Serotonin Toxicity Criteria highlights five potential scenarios in which a patient can be diagnosed with serotonin syndrome.

**FIGURE 1:**
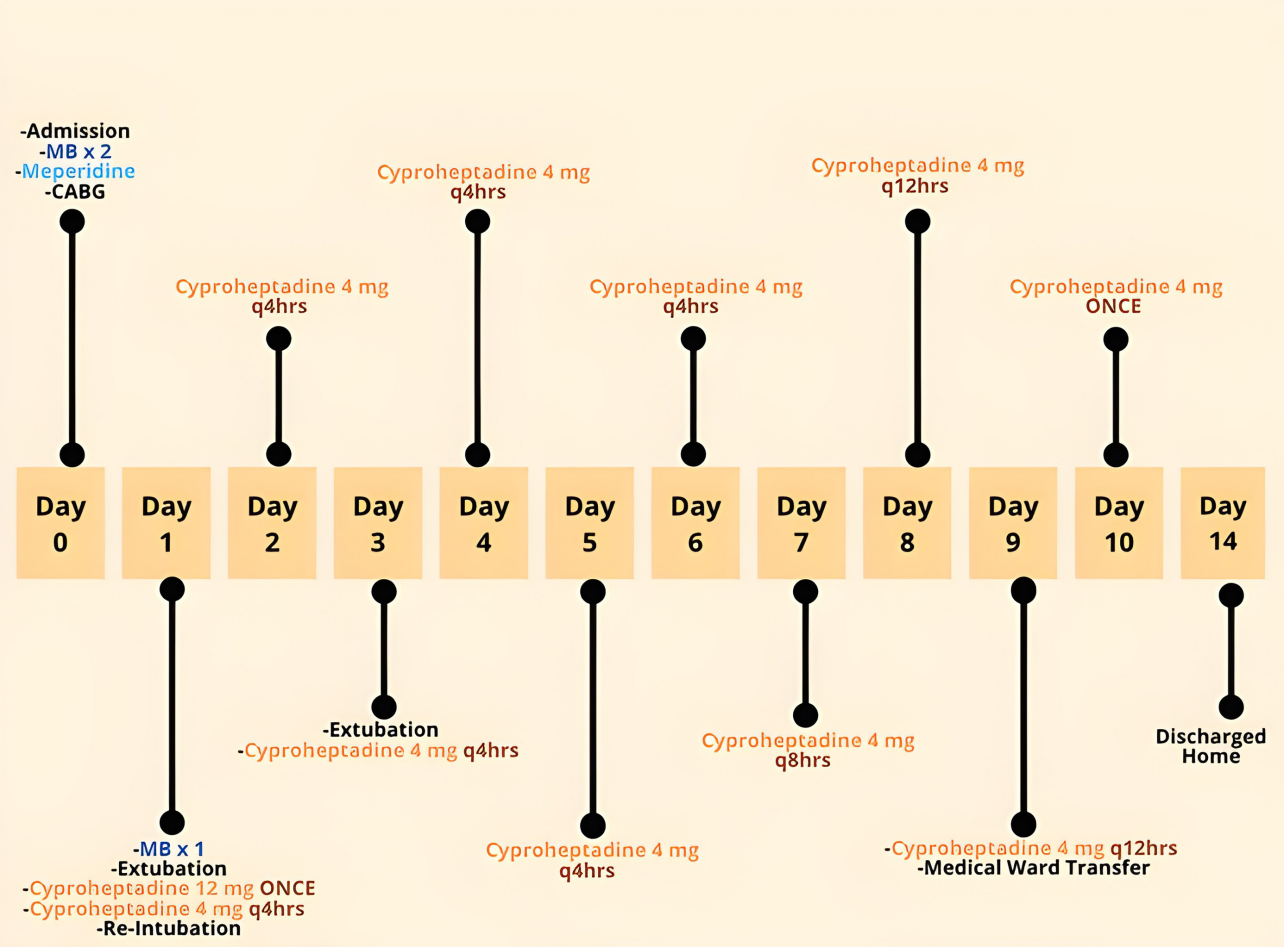
Event time line. This figure shows the progression of events from symptom presentation to patient discharge.

**FIGURE 2:**
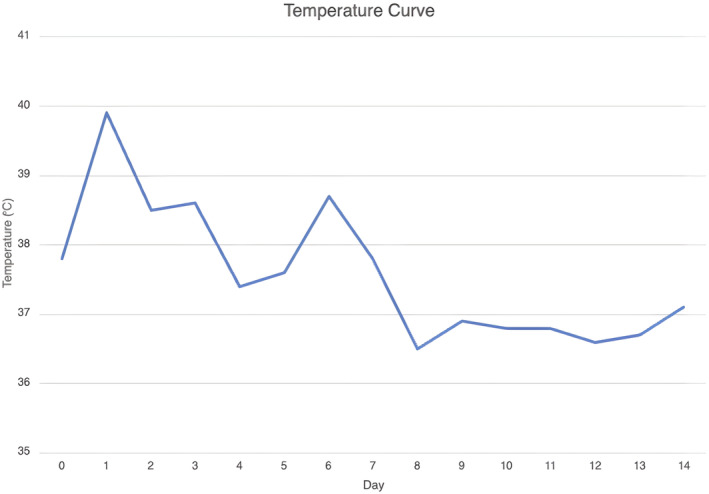
Temperature curve. This figure shows the temperature variability of the patient for 14 days postsurgery.

The patient was emergently reintubated on POD#1 and sedated with a midazolam and dexmedetomidine infusion to treat agitation and muscle rigidity. Cyproheptadine (serotonin antagonist) was started on POD#1 with 12 mg via the nasogastric tube. This was followed by 4 mg every 4 h starting on POD#1, then decreased to 4 mg every 8 h on POD#7, then decreased to 4 mg every 12 h on POD#8, and then a onetime dose of 4 mg was given on POD#10 (Figure 1). Treatment duration with cyproheptadine lasted a total of 10 days. External cooling was initiated for hyperthermia. The temperature curve of the patient is presented in Figure 2. Steps were taken to avoid medications with serotonergic properties.

Of note, midazolam infusion was discontinued on POD #3, which was the same day the patient was extubated, and dexmedetomidine infusion was discontinued on POD#4. The patient was transferred from the intensive care unit to the medical floor on POD#9. The patient was discharged home on POD#14 without any permanent impairment and was restarted on bupropion, based on a psychiatric consult on POD#13, with a plan to restart fluoxetine. The cyproheptadine dosing regimen caused no side effects in our patient.

## 3 DISCUSSION

As previously discussed, SS is not easy to diagnose as patients can present extremely variable, both in symptoms and in severity. In addition, there are other differential diagnoses that present very similarly that SS can be mistaken for. Neuroleptic malignant syndrome and malignant hyperthermia are two other drug‐induced syndromes that were the most relevant in this patient, especially in the postoperative setting. Table 2 highlights how similar these drug‐induced syndromes can present in patients.^5^, ^6^

**TABLE 2:** Differentiating drug‐induced syndromes.^a^

	Serotonin syndrome	Neuroleptic malignant syndrome	Malignant hyperthermia
Agitation	✔	✔	✔
Confusion	✔	✔	✔
Hyperthermia	✔	✔	✔
Tachycardia	✔	✔	✔
Hypertension	✔	✔	✔
Diaphoresis	✔	✔	✔
Hyperreflexia	✔	✖	✖
Tremor	✔	✔	✖
Mydriasis	✔	✖	✖
Increased bowel sounds	✔	✖	✖
Muscle rigidity	✔	✔	✔
Ocular/muscular clonus	✔	✖	✖

^a^ Serotonin syndrome, neuroleptic malignant syndrome, and malignant hyperthermia are three drug‐induced syndromes that have very similar signs and symptoms, making them difficult to distinguish among them as a primary diagnosis for a patient.

Our patient most likely had a drug–drug interaction between fluoxetine (SSRI), methylene blue (MAOI), and bupropion (inhibitor of reuptake of norepinephrine and dopamine). Even though we recognize that meperidine, an opioid with serotonergic activity, was administered to the patient, it is unlikely that it played a huge role because the symptoms started a day after meperidine was given and it is generally recommended to monitor a patient for symptoms for about 4 h if a serotonergic agent must be given while a patient is on methylene blue.^7^ In addition, data on meperidine causing SS is limited to case reports and none, to our knowledge, are in combination specifically with methylene blue.^8^, ^9^, ^10^, ^11^ Although discontinued 2 days prior to surgery, fluoxetine has a long half‐life. The parent drug has a half‐life of 3–4 days, while its active metabolite, norfluoxetine, has a half‐life of 9 days. This is why it is recommended to allow 5 weeks to elapse between discontinuing fluoxetine and the initiation of an MAOI, unless emergently required.^12^ Methylene blue is a highly potent reversible MAOI. Its half‐life is not as well‐defined as fluoxetine, but it is shorter at about 5 h.^7^ In treating vasoplegia, methylene blue may inhibit the effects of increased nitric oxide stimulation and guanylate cyclase activity, counteracting the uncontrolled vasodilation.^4^ However, there was likely dose accumulation that occurred as three doses of methylene blue were given within a 24‐h period. A similar case report in which a patient was on sertraline 100 mg daily and received methylene blue for vasoplegia after coronary artery bypass graft surgery, showed that SS can occur even with just one dose of methylene blue.^13^ By contrast, bupropion has no direct/indirect effect on serotonin, despite prior case reports stating this. Nonetheless, bupropion use is contraindicated with methylene blue as the combination can lead to hypertension, making autonomic instability that occurs with SS harder to treat.^14^

Cyproheptadine dosing recommendation is to give 12 mg orally once followed by 2 mg every 2 h until response with maintenance dosing being 4–8 mg every 6 h (maximum dose 32 mg/day).^1^, ^15^ Although initial dosing is clear, there is no strong evidence about how to wean doses once SS is treated. The reason why initial dosing is clear is that treatment of SS in adults may require 12–32 mg of cyproheptadine in a 24‐h period because, at this dose, 85%–95% of serotonin receptors are bound by cyproheptadine.^16^ The concern with rapid discontinuation of cyproheptadine is the potential recurrence of SS. Our patient received the same initial dose but was given 4 mg every 4 h as maintenance dosing. A single institution case series assessed 28 patients who received cyproheptadine for SS treatment. Of those patients, initial dosing ranged from 0 to 44 mg (12 mg—our case), cumulative dose ranged from 4 to 116 mg (188 mg—our case), treatment duration ranged from 1 to 7 days (10 days—our case), and an average daily dose of 18.4 mg/day (18.8 mg/day—our case). It highlighted the variety of dosing regimens but did not discuss weaning or titrating of cyproheptadine.^17^ Another case series reported rapid discontinuation of cyproheptadine upon symptom resolution and/or adverse event occurrence in four cases. The adverse events that occurred with administration of cyproheptadine included blurred vision and transient mydriasis, which can be used as an indicator that the SS has been resolved and treated.^18^ Our case is an example of a dosing regimen that may be replicated in other patients who present similarly to our patient.

There are several methods to minimize the risk of SS. Medication reconciliation is a great way to find serotonergic medications. The more medications that act on serotonin receptors, the more likely it is for SS to occur. Frequency and duration of the medications will affect the risk assessment. Performing drug interaction checks can help identify agents that are not commonly thought of as serotonergic. Early discontinuation of interacting medications or limiting the number of doses of methylene blue can reduce the risk. Treatment with vitamin B12 (hydroxocobalamin) is an alternative therapy for vasoplegia. Hydroxocobalamin has been used in the treatment of smoke inhalation and cyanide poisoning. The proposed mechanism of action in vasoplegia is the inhibition of the binding and excretion of nitric oxide and H2S (vasodilators).^19^ There are no known contraindications to the use of hydroxocobalamin and it represents a viable treatment option for patients that are refractory or intolerant to methylene blue.

## 4 CONCLUSION

We describe a case in which a drug–drug interaction of several medications was associated with SS. Based on available literature, there is no standardized method of weaning cyproheptadine doses when it is being used for the treatment of SS. This case provides a dosing regimen that could be used for other patients that present similarly. This also highlights the importance of medication reconciliation, drug–drug interactions, pharmacokinetics, and knowledge of drug‐induced syndromes to facilitate prompt treatment.

## AUTHOR CONTRIBUTIONS

**Ahmed Nagy:** Conceptualization; data curation; formal analysis; funding acquisition; investigation; methodology; project administration; resources; software; supervision; validation; visualization; writing – original draft; writing – review and editing. **Aishah Nasir:** Conceptualization; data curation; formal analysis; funding acquisition; investigation; methodology; project administration; resources; software; supervision; validation; visualization; writing – original draft; writing – review and editing. **Mahfujul Haque:** Conceptualization; data curation; formal analysis; funding acquisition; investigation; methodology; resources; software; supervision; validation; visualization; writing – original draft; writing – review and editing. **Ramzan Judge:** Conceptualization; data curation; formal analysis; funding acquisition; investigation; methodology; project administration; resources; software; supervision; validation; visualization; writing – original draft; writing – review and editing. **Joseph Lee:** Resources; software; supervision; validation; visualization; writing – original draft; writing – review and editing.

## CONFLICT OF INTEREST STATEMENT

The authors declare no conflicts of interest.

## CONSENT

Written informed consent was obtained from the patient to publish this report in accordance with the journal's patient consent policy.

## Data Availability

The data that support the findings of this study are available from the corresponding author upon reasonable request.
